# Roles of oral anticoagulant use on the risk of 28-day mortality and in-hospital mortality in patients with acute respiratory distress syndrome

**DOI:** 10.3389/fphar.2025.1565312

**Published:** 2025-05-14

**Authors:** Jiayang Huang, Huijie An, Lin Cheng, Wangsheng Li, Ke Zhang, Dan Su

**Affiliations:** ^1^ Department of Pharmacy, Shenzhen People’s Hospital (The Second Clinical Medical College, Jinan University, The First Affiliated Hospital, Southern University of Science and Technology), Shenzhen, China; ^2^ Department of Pharmacy, General Hospital of Southern Theatre Command, PLA, Guangzhou, China; ^3^ Department of Pharmacy, The Third People’s Hospital of Longgang District Shenzhen, Shenzhen, China; ^4^ Department of Pharmacy, Shaoxing People’s Hospital, Shaoxing, China; ^5^ Health Management Center, Shenzhen University General Hospital, Shenzhen, China

**Keywords:** oral anticoagulant, ARDS, 28-day mortality, in-hospital mortality, warfain

## Abstract

**Aim:**

This study was to investigate the association between oral anticoagulant use and 28-day mortality and in-hospital mortality in patients with acute respiratory distress syndrome (ARDS).

**Methods:**

A total of 1754 ARDS patients were identified in database from 2008 to 2022 in this cohort study. Univariable and multivariable cox regression models were applied to assess the associations of oral anticoagulant use with the risk of 28-day mortality and in-hospital mortality. Propensity score matching (PSM) was performed in ARDS patients according to whether they were taking oral anticoagulants or not to control potential bias. Subgroup analysis was performed according to severity of ARDS (mild, moderate, and severe), and comorbidities (atrial fibrillation, sepsis, and AKI). Hazards ratio (HR) and respective confidence interval (CI) were presented.

**Results:**

In total, 7758 patients not receiving oral anticoagulant and 905 patients receiving oral anticoagulant. The reduced risk of 28-day mortality in ARDS patients was identified in those undergoing oral anticoagulant use (HR = 0.32, 95%CI: 0.24–0.44). Oral anticoagulant use was associated with reduced risk of in-hospital mortality (HR = 0.27, 95%CI: 0.20–0.37). After adjusting for the respective confounding factors, the associations of Warfarin with decreased risk of 28-day and in-hospital mortality were not significant (*P* > 0.05).

**Conclusion:**

Oral anticoagulant was related to decreased risk of 28-day/in-hospital mortality in patients with ARDS. Warfarin and novel oral anticoagulants showed no significant difference on 28-day/in-hospital mortality in patients with ARDS.

## Introduction

Acute respiratory distress syndrome (ARDS) is a clinical syndrome characterized by acute respiratory failure resulting from non-cardiogenic pulmonary edema (Bos and Ware, 2022). ARDS is among the most prevalent conditions in intensive care units and constitutes a significant cause of short-term mortality, imposing an enormous disease burden globally (Meyer et al., 2021). ARDS is defined by inflammatory lung injury and is associated with a global mortality rate approaching 40% (Bellani et al., 2016). To identify factors associated with short-term mortality was essential for improving the prognosis of these patients.

Pulmonary coagulopathy is a vital participant in the pathophysiology of ARDS, which is characterized by activated coagulation and decreased fibrinolysis (Camprubí-Rimblas et al., 2018; Nieuwenhuizen et al., 2009). Previous evidence indicated that coagulation function is an important factor affecting organ failure and in-hospital mortality in ARDS patients, which may be related to the increased level of inflammation associated with hypercoagulability (Gando et al., 2020; Li and Pan, 2024). Thus, the function of anticoagulants has been explored. Studies have found that early use of heparin may be associated with a reduced risk of short-term death in patients with ARDS (Xiao et al., 2024), but the conclusions are not consistent (Hofstra et al., 2012). Previous evidence also indicated that the impact of chronic oral anticoagulation therapy on the clinical outcomes of hospitalized patients with COVID-19 remains a subject of ongoing debate (Flam et al., 2021; Denas et al., 2021). Russo et al. revealed that oral anticoagulation therapy did not influence the risk of ARDS or death in patients hospitalized with COVID-19 (Russo et al., 2022). Wenzler et al. identified that apixaban for therapeutic anticoagulation was safe and efficacious in ICU patients with severe COVID-19 respiratory disease (Wenzler et al., 2020). Thus, the role of oral anticoagulation use on the prognosis of ARDS patients still require investigation. In addition, due to the need for injection of heparin and its possible adverse reactions such as heparin-induced thrombocytopenia (HIT), oral anticoagulants are gradually being used in critically ill patients (Wahab et al., 2021). In critically ill patients with both atrial fibrillation and sepsis or atrial fibrillation and acute kidney injury, the use of oral anticoagulants was found to be significantly associated with reduced 30-day mortality risk, whereas no significant difference was found between warfarin and novel oral anticoagulants (Ge et al., 2024; Bo et al., 2023). To date, no studies have investigated the relationship between the use of oral anticoagulants and the short-term prognosis of patients with ARDS.

The objectives of this study were to investigate the association between oral anticoagulant use and 28-day mortality and in-hospital mortality in patients with ARDS. The effects of warfarin and novel oral anticoagulants on short-term mortality in patients with ARDS were also compared.

## Materials and methods

### Study design and population

A total of 9359 participants ≥18 years diagnosed as ARDS were identified in database from 2008 to 2022 in this cohort study. The MIMIC-IV database consists of comprehensive and high-quality medical records from patients admitted to the intensive care units of Beth Israel Deaconess Medical Center (Johnson et al., 2023). In our study, the included criteria were that 1) age ≥ 18 years, 2) diagnosis of ARDS at admission (Berlin criteria). The Berlin criteria for ARDS included acute onset, partial pressure of oxygen (PaO_2_)/fraction of inspired oxygen (FiO_2_) ≤300 mmHg, positive end-expiratory pressure (PEEP) ≥ 5 cm H_2_O, bilateral infiltrates on chest radiograph, and absence of heart failure. Mild, moderate, and severe ARDS were classified based on the PaO_2_/FiO_2_ ratios of the patients: mild (200 mmHg < PaO_2_/FiO_2_ ≤ 300 mmHg), moderate (100 mmHg < PaO_2_/FiO^2^ ≤ 200 mmHg), and severe (PaO_2_/FiO_2_ ≤ 100 mmHg) (Ranieri et al., 2012). The severity of ARDS was defined as mild 200 mmHg < PaO_2_/FIO_2_ ≤ 300 mmHg with PEEP or CPAP≥5 cm H_2_O, moderate 100 mmHg < PaO_2_/FIO_2_ ≤ 200 mmHg with PEEP ≥5 cm H_2_O, and severe PaO2/FIO2 ≤ 100 mmHg with PEEP ≥5 cm H_2_O (Ranieri et al., 2012). Patients were excluded if they stayed in ICU < 24 h, and missing the survival information.

### Potential covariables and definitions

Age (years), gender (female or male), ethnicity (White, Black or other), weight (kg), ARDS severity (mild, moderate, or severe), atrial fibrillation or not, diabetes or not, cerebral infarction or not, pneumonia or not, sepsis or not, acute kidney injury (AKI) or not, heart rate (bpm), mean arterial pressure (mmHg), respiratory rate (insp/min), temperature (Deg.C), sepsis related organ failure assessment (SOFA), Charlson comorbidity index (CCI), white blood cell count (K/uL), red blood cell volume distribution width (RDW) (%), hematocrit (%), creatinine (mg/dL), blood urea nitrogen.

(BUN) (mg/dL), glucose (mg/dL), anion gap (mEq/L), calcium (mg/dL), oxygen saturation (SpO_2_) (%), partial pressure of carbon dioxide in arterial blood (PaCO_2_) (mmHg), partial thromboplastin time (PTT) (sec), mechanical ventilation duration (<48 h or ≥48 h), using vasopressors or not, using antibiotics or not, and using renal replacement therapy (RRT) or not were potential confounding factors. All the data were analyzed used the first measurement during 24 h ICU admission.

Mechanical ventilation were calculated based on parameters including peak inspiratory pressure (Ppeak), positive end-expiratory pressure, measured respiratory rate, and tidal volume. Atrial fibrillation was diagnosed based on International Classification of Diseases (ICD)-9 codes (42,731, and 42,732), and ICD-10 with first three digits of I48. Diabetes was diagnosed based on ICD-9 with first three digits of 250, and ICD-10 with first three digits of E10, E11, E12, E13, and E14. Cerebral infarction was diagnosed based on ICD-9 (433.01-433.91, and 434.01-91), and ICD-10 (I63). Pneumonia was diagnosed based on ICD-9 with first three digits of 480 to 486, and ICD10 with first three digits of J12 to J18.

### Main variable

Data on anticoagulant drugs were extracted based on the Prescription list in MIMIC-IV database. Patients with the use oral anticoagulant drugs including Warfarin, Apixaban, Rivaroxaban, and Dabigatran during the time from ICU admission to ICU discharge were regarded to have oral anticoagulant use. Patients used Apixaban, Rivaroxaban, or Dabigatran were regraded to use novel oral anticoagulant drugs.

### Outcome variables

The 28-day mortality and in-hospital mortality were outcomes. The follow-up of 28-day mortality was ended if the patients died within 28 days or follow-up to 28-day if the patients survived. The median follow-up time of 28-day mortality was 28 days. In-hospital mortality was followed-up to the time of in-hospital death or time of discharge from the hospital. The median follow-up time of in-hospital mortality was 7.34 days.

### Statistical analysis

The measurement data with normal distribution were described by Mean ± standard deviation (Mean ± SD), and the t-test was used to compare the differences between groups. The median and quartiles were used to describe the distribution of measurement data that did not obey the normal distribution, and the Wilcoxon rank sum test was used to compare the differences between group. The enumeration data were described as number and percentage, and the chi-square test was used to compare the differences between groups. Missing values were presented in Supplementary Table S1. The missing variables were filled by multiple interpolation method, and data were compared before and after missing value imputation (Supplementary Table S2). Univariable cox regression model was constructed to evaluate the covariables associated with 28-day mortality and in-hospital mortality, respectively. Univariable and multivariable cox regression models were applied to assess the associations of oral anticoagulant use with the risk of 28-day mortality and in-hospital mortality. Propensity score matching (PSM) was performed in ARDS patients according to whether they were taking oral anticoagulants or not to control potential bias. Subgroup analysis was performed according to severity of ARDS (mild, moderate, and severe), and comorbidities (atrial fibrillation, sepsis, and AKI). Hazards ratio (HR) and respective confidence interval (CI) were presented. Kaplan-Meier curves were plotted to compare the survival probability of patients receiving oral anticoagulant or not. Data analysis was conducted using R [R version 4.4.0 (2024-04–24 ucrt)]. *P* < 0.05 was regarded to be statistically significant.

## Results

### Comparisons of characteristics of participants receiving oral anticoagulant or not receiving oral anticoagulant

**FIGURE 1:**
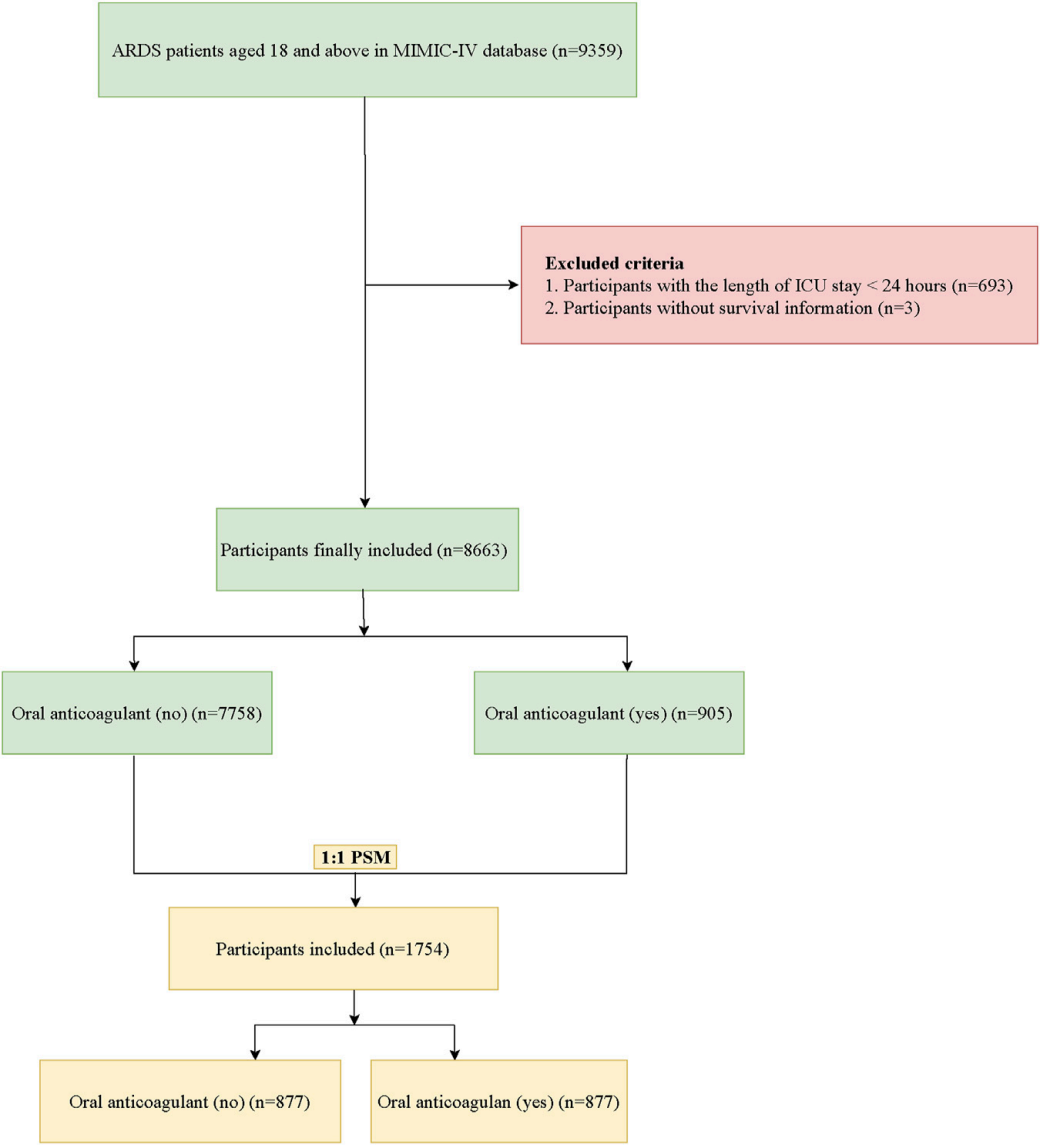
The screening process of participants.

In total, 9359 ARDS patients aged ≥18 years were identified in the MIMIC-IV database. Among them, patients who stayed in ICU < 24 h were excluded. Also, those without survival information were not included. Finally, 8,663 participants were included with 7758 patients not receiving oral anticoagulant and 905 patients receiving oral anticoagulant. After PSM, 1754 participants were analyzed. The screening process of participants was shown Figure 1.

**FIGURE 2:**
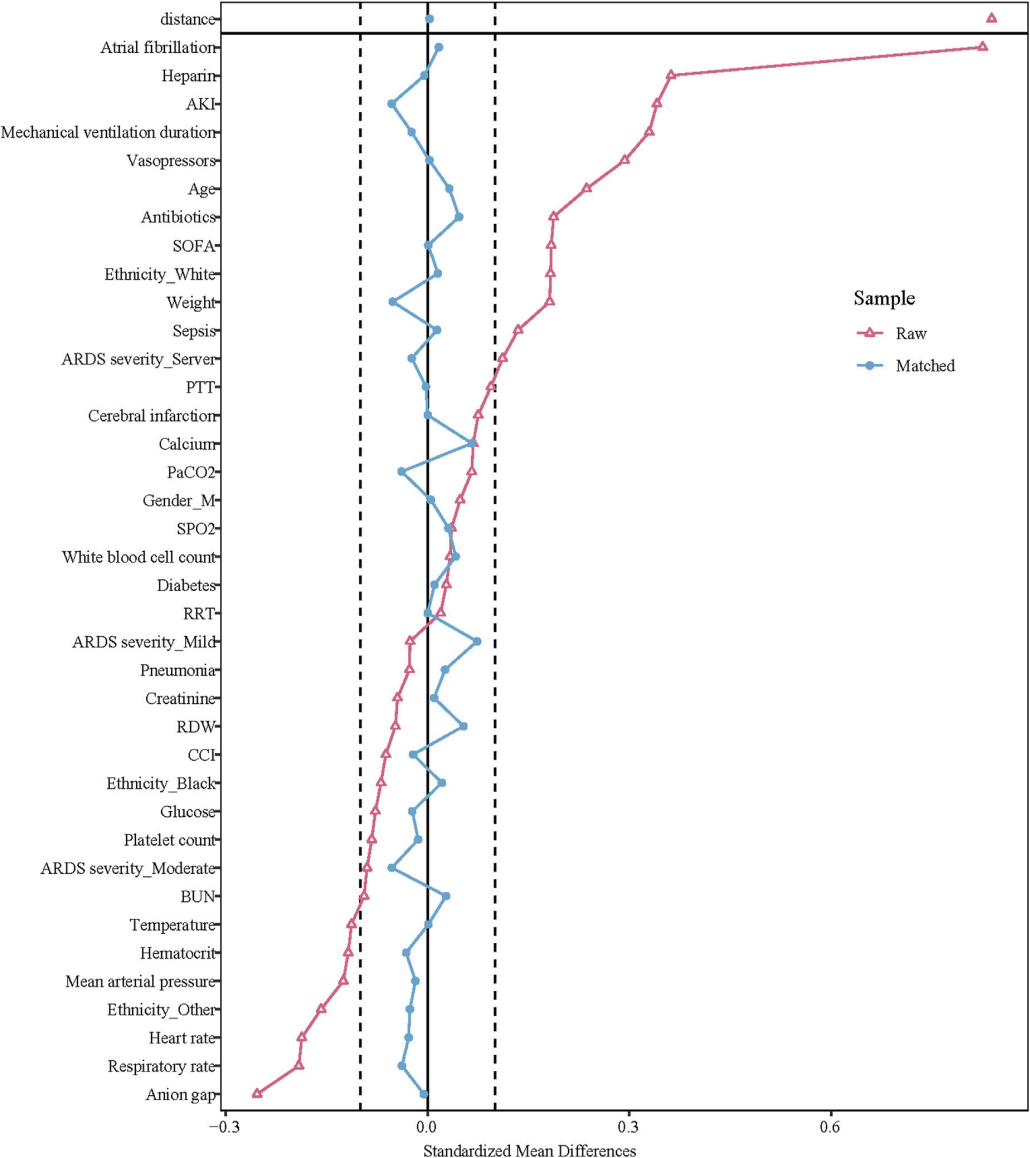
The SMD of data before PSM and after PSM.

Before PSM, the mean age of the oral anticoagulant group was older than the non-oral anticoagulant group (64.59 years vs. 61.29 years). The percentage of atrial fibrillation patients was higher in the oral anticoagulant group relative to the non-oral anticoagulant group (62.98% vs. 23.14%). The percentage of cerebral infarction patients was higher in the oral anticoagulant group compared to the non-oral anticoagulant group (0.88% vs. 0.18%). The median SOFA score in the oral anticoagulant group was higher than the non-oral anticoagulant group (3.00 vs. 2.00). After PSM, the data in the oral anticoagulant group and the non-oral anticoagulant group were equilibrium. More information of the characteristics was exhibited in Table 1. The standardized mean difference (SMD) of data before PSM and after PSM was presented in Figure 2.

**TABLE 1:** Characteristics of participant with or without oral anticoagulant use before and after PSM.

Variables	Before PSM					After PSM				
	Total (n = 8,663)	Oral anticoagulant:No (n = 7758)	Oral anticoagulant:Yes (n = 905)	Statistics	*P*	Total (n = 1754)	Oral anticoagulant:No (n = 877)	Oral anticoagulant:Yes (n = 877)	Statistics	*P*
Age (years), mean ± SD	61.63 ± 15.16	61.29 ± 15.26	64.59 ± 13.97	t = −6.66	<0.001	64.33 ± 14.14	64.11 ± 14.24	64.56 ± 14.05	t = −0.67	0.506
Gender, n (%)				χ^2^ = 1.833	0.176				χ^2^ = 0.010	0.920
Female	3,077 (35.52)	2,774 (35.76)	303 (33.48)			592 (33.75)	297 (33.87)	295 (33.64)		
Male	5,586 (64.48)	4,984 (64.24)	602 (66.52)			1,162 (66.25)	580 (66.13)	582 (66.36)		
Ethnicity, n (%)				χ^2^ = 24.142	<0.001				χ^2^ = 0.440	0.803
White	5,461 (63.04)	4,823 (62.17)	638 (70.50)			1,222 (69.67)	608 (69.33)	614 (70.01)		
Black	548 (6.33)	503 (6.48)	45 (4.97)			86 (4.90)	41 (4.68)	45 (5.13)		
Other	2,654 (30.64)	2,432 (31.35)	222 (24.53)			446 (25.43)	228 (26.00)	218 (24.86)		
Weight (kg), mean ± SD	87.79 ± 25.31	87.29 ± 25.12	92.10 ± 26.52	t = −5.19	<0.001	89.00 (75.34, 105.23)	89.00 (76.43, 104.78)	88.81 (74.84, 106.00)	Z = 0.529	0.597
ARDS severity, n (%)				χ^2^ = 12.072	0.002				χ^2^ = 2.570	0.277
Mild	3,286 (37.93)	2,953 (38.06)	333 (36.80)			619 (35.29)	294 (33.52)	325 (37.06)		
Moderate	2,736 (31.58)	2,483 (32.01)	253 (27.96)			515 (29.36)	268 (30.56)	247 (28.16)		
Severe	2,641 (30.49)	2,322 (29.93)	319 (35.25)			620 (35.35)	315 (35.92)	305 (34.78)		
Atrial fibrillation, n (%)				χ^2^ = 648.340	<0.001				χ^2^ = 0.118	0.731
No	6,298 (72.70)	5,963 (76.86)	335 (37.02)			677 (38.60)	342 (39.00)	335 (38.20)		
Yes	2,365 (27.30)	1795 (23.14)	570 (62.98)			1,077 (61.40)	535 (61.00)	542 (61.80)		
Diabetes, n (%)				χ^2^ = 0.633	0.426				χ^2^ = 0.045	0.832
No	6,290 (72.61)	5,643 (72.74)	647 (71.49)			1,256 (71.61)	630 (71.84)	626 (71.38)		
Yes	2,373 (27.39)	2,115 (27.26)	258 (28.51)			498 (28.39)	247 (28.16)	251 (28.62)		
Cerebral infarction, n (%)				-	0.001				χ^2^ = 0.000	1.000
No	8,641 (99.75)	7744 (99.82)	897 (99.12)			1742 (99.32)	871 (99.32)	871 (99.32)		
Yes	22 (0.25)	14 (0.18)	8 (0.88)			12 (0.68)	6 (0.68)	6 (0.68)		
Pneumonia, n (%)				χ^2^ = 0.578	0.447				χ^2^ = 0.295	0.587
No	6,885 (79.48)	6,157 (79.36)	728 (80.44)			1,413 (80.56)	711 (81.07)	702 (80.05)		
Yes	1778 (20.52)	1,601 (20.64)	177 (19.56)			341 (19.44)	166 (18.93)	175 (19.95)		
Sepsis, n (%)				χ^2^ = 15.124	<0.001				χ^2^ = 0.083	0.774
No	5,172 (59.70)	4,686 (60.40)	486 (53.70)			944 (53.82)	475 (54.16)	469 (53.48)		
Yes	3,491 (40.30)	3,072 (39.60)	419 (46.30)			810 (46.18)	402 (45.84)	408 (46.52)		
AKI, n (%)				χ^2^ = 51.673	<0.001				χ^2^ = 1.320	0.251
No	1,461 (16.86)	1,385 (17.85)	76 (8.40)			139 (7.92)	63 (7.18)	76 (8.67)		
Yes	7202 (83.14)	6,373 (82.15)	829 (91.60)			1,615 (92.08)	814 (92.82)	801 (91.33)		
Heart rate (bpm), mean ± SD	88.78 ± 19.79	89.12 ± 20.01	85.84 ± 17.50	t = 5.25	<0.001	86.13 ± 18.23	86.38 ± 18.87	85.88 ± 17.57	t = 0.57	0.569
Mean arterial pressure (mmHg), mean ± SD	83.90 ± 17.43	84.12 ± 17.54	82.06 ± 16.40	t = 3.55	<0.001	82.34 ± 16.88	82.48 ± 17.40	82.19 ± 16.35	t = 0.37	0.711
Respiratory rate (insp/min), M (Q_1_, Q_3_)	17.00 (15.00, 22.00)	18.00 (15.00, 22.00)	16.00 (14.00, 20.00)	Z = −5.975	<0.001	16.00 (14.00, 20.00)	16.00 (14.00, 21.00)	16.00 (14.00, 20.00)	Z = 1.599	0.110
Temperature (Deg.C), mean ± SD	36.57 ± 1.04	36.58 ± 1.05	36.47 ± 0.93	t = 3.18	0.002	36.47 ± 1.01	36.47 ± 1.09	36.47 ± 0.93	t = −0.02	0.985
SOFA, M (Q_1_, Q_3_)	2.00 (1.00, 4.00)	2.00 (0.00, 4.00)	3.00 (1.00, 5.00)	Z = 6.106	<0.001	3.00 (1.00, 4.00)	2.00 (1.00, 4.00)	3.00 (1.00, 5.00)	Z = −0.533	0.594
CCI, M (Q_1_, Q_3_)	2.00 (1.00, 3.00)	2.00 (1.00, 3.00)	2.00 (1.00, 3.00)	Z = 0.120	0.905	2.00 (1.00, 3.00)	2.00 (1.00, 3.00)	2.00 (1.00, 3.00)	Z = −0.596	0.551
White blood cell count (K/uL), M (Q_1_, Q_3_)	12.50 (8.90, 16.80)	12.40 (8.80, 16.80)	12.90 (9.70, 16.90)	Z = 2.760	0.006	12.70 (9.30, 16.90)	12.40 (9.00, 16.80)	12.90 (9.70, 16.90)	Z = −1.940	0.052
Platelet count (K/uL), M (Q_1_, Q_3_)	169.00 (124.00, 233.00)	171.00 (124.00, 234.00)	159.00 (122.00, 220.00)	Z = −2.496	0.013	160.00 (121.00, 224.00)	164.00 (118.00, 228.00)	159.00 (123.00, 222.00)	Z = 0.323	0.747
RDW (%), mean ± SD	14.51 ± 2.04	14.52 ± 2.08	14.44 ± 1.74	t = 1.34	0.181	14.37 ± 1.74	14.32 ± 1.75	14.42 ± 1.73	t = −1.11	0.265
Hematocrit (%), mean ± SD	32.82 ± 6.91	32.91 ± 6.92	32.11 ± 6.74	t = 3.28	0.001	32.25 ± 6.75	32.36 ± 6.81	32.14 ± 6.69	t = 0.67	0.504
Creatinine (mg/dL), M (Q_1_, Q_3_)	0.90 (0.70, 1.30)	0.90 (0.70, 1.30)	0.90 (0.70, 1.30)	Z = −0.776	0.438	0.90 (0.70, 1.30)	0.90 (0.70, 1.20)	0.90 (0.70, 1.30)	Z = 0.259	0.795
BUN (mg/dL), M (Q_1_, Q_3_)	17.00 (13.00, 26.00)	17.00 (13.00, 26.00)	17.00 (12.00, 24.00)	Z = −1.640	0.101	17.00 (13.00, 24.00)	17.00 (13.00, 24.00)	17.00 (12.00, 25.00)	Z = 0.100	0.920
Glucose (mg/dL), M (Q_1_, Q_3_)	140.00 (115.00, 176.00)	140.00 (115.00, 177.00)	142.00 (116.00, 171.00)	Z = 0.031	0.975	143.00 (117.00, 172.00)	143.00 (118.00, 173.00)	142.00 (116.00, 172.00)	Z = 0.657	0.511
Anion gap (mEq/L), M (Q_1_, Q_3_)	13.00 (11.00, 16.00)	13.00 (11.00, 16.00)	12.00 (10.00, 15.00)	Z = −5.948	<0.001	12.00 (10.00, 15.00)	12.00 (10.00, 15.00)	12.00 (10.00, 15.00)	Z = −0.289	0.773
Calcium (mg/dL), mean ± SD	8.11 ± 0.94	8.10 ± 0.95	8.16 ± 0.83	t = −1.90	0.058	8.13 ± 0.83	8.10 ± 0.83	8.16 ± 0.83	t = −1.38	0.167
SpO_2_ (%), mean ± SD	97.49 ± 4.38	97.48 ± 4.41	97.62 ± 4.10	t = −1.00	0.317	97.54 ± 4.28	97.48 ± 4.41	97.60 ± 4.15	t = −0.62	0.536
PaCO_2_ (mmHg), mean ± SD	43.43 ± 11.40	43.35 ± 11.35	44.13 ± 11.86	t = −1.94	0.052	44.38 ± 11.63	44.61 ± 11.33	44.15 ± 11.92	t = 0.83	0.407
PTT (sec), M (Q_1_, Q_3_)	30.40 (27.00, 36.40)	30.30 (27.00, 36.00)	31.50 (27.60, 38.50)	Z = 4.918	<0.001	31.10 (27.60, 38.00)	30.80 (27.50, 37.40)	31.50 (27.60, 38.50)	Z = −1.622	0.105
Mechanical ventilation duration (hours), n (%)				χ^2^ = 79.732	<0.001				χ^2^ = 0.250	0.617
<48	4,222 (48.74)	3,908 (50.37)	314 (34.70)			616 (35.12)	303 (34.55)	313 (35.69)		
≥48	4,441 (51.26)	3,850 (49.63)	591 (65.30)			1,138 (64.88)	574 (65.45)	564 (64.31)		
Vasopressors, n (%)				χ^2^ = 57.768	<0.001				χ^2^ = 0.003	0.957
No	3,344 (38.60)	3,100 (39.96)	244 (26.96)			485 (27.65)	243 (27.71)	242 (27.59)		
Yes	5,319 (61.40)	4,658 (60.04)	661 (73.04)			1,269 (72.35)	634 (72.29)	635 (72.41)		
Antibiotics, n (%)				χ^2^ = 20.573	<0.001				χ^2^ = 0.896	0.344
No	1,447 (16.70)	1,344 (17.32)	103 (11.38)			215 (12.26)	114 (13.00)	101 (11.52)		
Yes	7216 (83.30)	6,414 (82.68)	802 (88.62)			1,539 (87.74)	763 (87.00)	776 (88.48)		
Heparin, n (%)				χ^2^ = 177.990	<0.001				χ^2^ = 0.012	0.914
No	7420 (85.65)	6,778 (87.37)	642 (70.94)			1,280 (72.98)	639 (72.86)	641 (73.09)		
Yes	1,243 (14.35)	980 (12.63)	263 (29.06)			474 (27.02)	238 (27.14)	236 (26.91)		
RRT, n (%)				χ^2^ = 0.314	0.575				χ^2^ = 0.000	1.000
No	7790 (89.92)	6,981 (89.98)	809 (89.39)			1,564 (89.17)	782 (89.17)	782 (89.17)		
Yes	873 (10.08)	777 (10.02)	96 (10.61)			190 (10.83)	95 (10.83)	95 (10.83)		
In-hospital mortality, n (%)				χ^2^ = 81.901	<0.001				χ^2^ = 57.980	<0.001
Alive	7204 (83.16)	6,355 (81.92)	849 (93.81)			1,537 (87.63)	716 (81.64)	821 (93.61)		
Dead	1,459 (16.84)	1,403 (18.08)	56 (6.19)			217 (12.37)	161 (18.36)	56 (6.39)		
Follow-up of in-hospital mortality, M (Q_1_, Q_3_)	7.34 (4.69, 14.84)	7.21 (4.40, 14.49)	9.22 (5.99, 18.57)	Z = 9.462	<0.001	8.59 (5.34, 16.81)	8.07 (5.22, 15.16)	9.22 (5.95, 18.88)	Z = −3.932	<0.001
28-day mortality, n (%)				χ^2^ = 83.901	<0.001				χ^2^ = 50.707	<0.001
Alive	7138 (82.40)	6,293 (81.12)	845 (93.37)			1,538 (87.69)	720 (82.10)	818 (93.27)		
Dead	1,525 (17.60)	1,465 (18.88)	60 (6.63)			216 (12.31)	157 (17.90)	59 (6.73)		
Follow-up of 28-day mortality, M (Q_1_, Q_3_)	28.00 (28.00, 28.00)	28.00 (28.00, 28.00)	28.00 (28.00, 28.00)	Z = 9.380	<0.001	28.00 (28.00, 28.00)	28.00 (28.00, 28.00)	28.00 (28.00, 28.00)	Z = −7.184	<0.001

t: t-test, Z: Mann-Whitney U test, χ^2^: Chi-square test, -: Fisher exact, SD: standard deviation, M: Median, Q_1_: 1st Quartile, Q_3_: 3st Quartile, PSM: propensity score matching, ARDS: acute respiratory distress syndrome, AKI: acute kidney injury, SOFA: sepsis related organ failure assessment, CCI: Charlson comorbidity index, RDW: red blood cell volume distribution width, BUN: blood urea nitrogen, SpO_2_: oxygen saturation, PaCO_2_: partial pressure of carbon dioxide in arterial blood, PTT: partial thromboplastin time, RRT: renal replacement therapy.

### Association between oral anticoagulant use with the risk of 28-day and in-hospital mortality

As delineated in gender, ethnicity, ARDS severity, cerebral infarction, pneumonia, sepsis, AKI, heart rate, respiratory rate, SOFA, CCI, WBC, creatinine, BUN, glucose, anion gap, SpO_2_, PTT, mechanical ventilation duration, antibiotics, and RRT were confounding factors related to 28-day mortality (Table 2). Compared to ARDS patients without oral anticoagulant use, the risk of 28-day mortality might be reduced in those receiving oral anticoagulant use (HR = 0.35, 95%CI: 0.26–0.47). After adjusting for the confounding factors, the reduced risk of 28-day mortality in ARDS patients was identified in those undergoing oral anticoagulant use (HR = 0.32, 95%CI: 0.24–0.44).

**TABLE 2:** Potential confounding factors associated with 28-day mortality and in-hospital mortality (after PSM, n = 1754).

Variables	28-day mortality		In-hospital mortality	
	HR (95%CI)	*P*	HR (95%CI)	*P*
Age (years)	1.01 (1.00–1.02)	0.050	1.01 (1.01–1.02)	0.008
Gender				
Female	Ref		Ref	
Male	0.64 (0.49–0.84)	0.001	0.69 (0.52–0.90)	0.006
Ethnicity				
White	Ref		Ref	
Black	1.42 (0.80–2.51)	0.226	1.03 (0.59–1.80)	0.903
Other	1.46 (1.09–1.95)	0.011	1.20 (0.90–1.60)	0.214
Weight (kg)	1.00 (0.99–1.00)	0.110	1.00 (0.99–1.00)	0.659
ARDS severity				
Mild	Ref		Ref	
Moderate	1.72 (1.22–2.44)	0.002	1.36 (0.95–1.94)	0.090
Severe	1.56 (1.11–2.19)	0.011	1.29 (0.91–1.83)	0.156
Atrial fibrillation				
No	Ref		Ref	
Yes	1.11 (0.84–1.46)	0.478	1.19 (0.90–1.57)	0.214
Diabetes				
No	Ref		Ref	
Yes	0.89 (0.65–1.20)	0.438	0.87 (0.64–1.18)	0.367
Cerebral infarction				
No	Ref		Ref	
Yes	3.08 (1.14–8.27)	0.026	1.84 (0.59–5.75)	0.296
Pneumonia				
No	Ref		Ref	
Yes	2.40 (1.81–3.17)	<0.001	1.37 (1.03–1.82)	0.028
Sepsis				
No	Ref		Ref	
Yes	1.33 (1.02–1.73)	0.038	0.95 (0.72–1.25)	0.709
AKI				
No	Ref		Ref	
Yes	6.40 (2.05–19.99)	0.001	3.51 (1.12–11.01)	0.031
Heart rate (bpm)	1.02 (1.01–1.02)	<0.001	1.01 (1.01–1.01)	0.004
Mean arterial pressure (mmHg)	1.00 (1.00–1.01)	0.220	1.00 (0.99–1.00)	0.420
Respiratory rate (insp/min)	1.07 (1.06–1.09)	<0.001	1.05 (1.03–1.07)	<0.001
Temperature (Deg.C)	1.07 (0.93–1.22)	0.360	0.95 (0.84–1.07)	0.424
SOFA	1.10 (1.05–1.15)	<0.001	1.09 (1.04–1.14)	<0.001
CCI	1.27 (1.20–1.34)	<0.001	1.17 (1.10–1.24)	<0.001
White blood cell count (K/uL)	1.01 (1.01–1.02)	0.002	1.01 (1.01–1.02)	0.008
Platelet count (K/uL)	1.00 (1.00–1.00)	0.193	1.00 (1.00–1.00)	0.445
RDW (%)	1.30 (1.24–1.38)	<0.001	1.22 (1.16–1.29)	<0.001
Hematocrit (%)	1.02 (1.00–1.04)	0.078	1.01 (0.99–1.03)	0.342
Creatinine (mg/dL)	1.21 (1.12–1.29)	<0.001	1.11 (1.03–1.19)	0.005
BUN (mg/dL)	1.02 (1.02–1.03)	<0.001	1.02 (1.01–1.02)	<0.001
Glucose (mg/dL)	1.01 (1.01–1.01)	<0.001	1.00 (1.00–1.00)	0.054
Anion gap (mEq/L)	1.11 (1.09–1.13)	<0.001	1.08 (1.06–1.10)	<0.001
Calcium (mg/dL)	0.88 (0.75–1.04)	0.129	0.95 (0.82–1.10)	0.506
SPO2 (%)	0.96 (0.94–0.98)	<0.001	0.98 (0.96–1.01)	0.131
PaCO2 (mmHg)	1.00 (0.99–1.01)	0.839	1.00 (0.99–1.01)	0.490
PTT (sec)	1.01 (1.01–1.02)	<0.001	1.01 (1.01–1.01)	<0.001
Mechanical ventilation duration (hours)				
<48	Ref		Ref	
≥48	1.96 (1.42–2.71)	<0.001	0.93 (0.64–1.34)	0.697
Vasopressors				
No	Ref		Ref	
Yes	1.27 (0.93–1.75)	0.135	1.32 (0.96–1.83)	0.090
Antibiotics				
No	Ref		Ref	
Yes	0.69 (0.48–0.99)	0.045	0.85 (0.59–1.24)	0.407
Heparin				
No	Ref		Ref	
Yes	1.12 (0.84–1.51)	0.440	0.94 (0.70–1.27)	0.704
RRT				
No	Ref		Ref	
Yes	3.88 (2.90–5.21)	<0.001	2.23 (1.67–2.99)	<0.001

Ref: reference, HR: hazard ratio, CI: confidence interval, PSM: propensity score matching, ARDS: acute respiratory distress syndrome, AKI: acute kidney injury, SOFA: sepsis related organ failure assessment, CCI: charlson comorbidity index, RDW: red blood cell volume distribution width, BUN: blood urea nitrogen, SpO2: oxygen saturation, PaCO2: partial pressure of carbon dioxide in arterial blood, PTT: partial thromboplastin time, RRT: renal replacement therapy.

**FIGURE 3:**
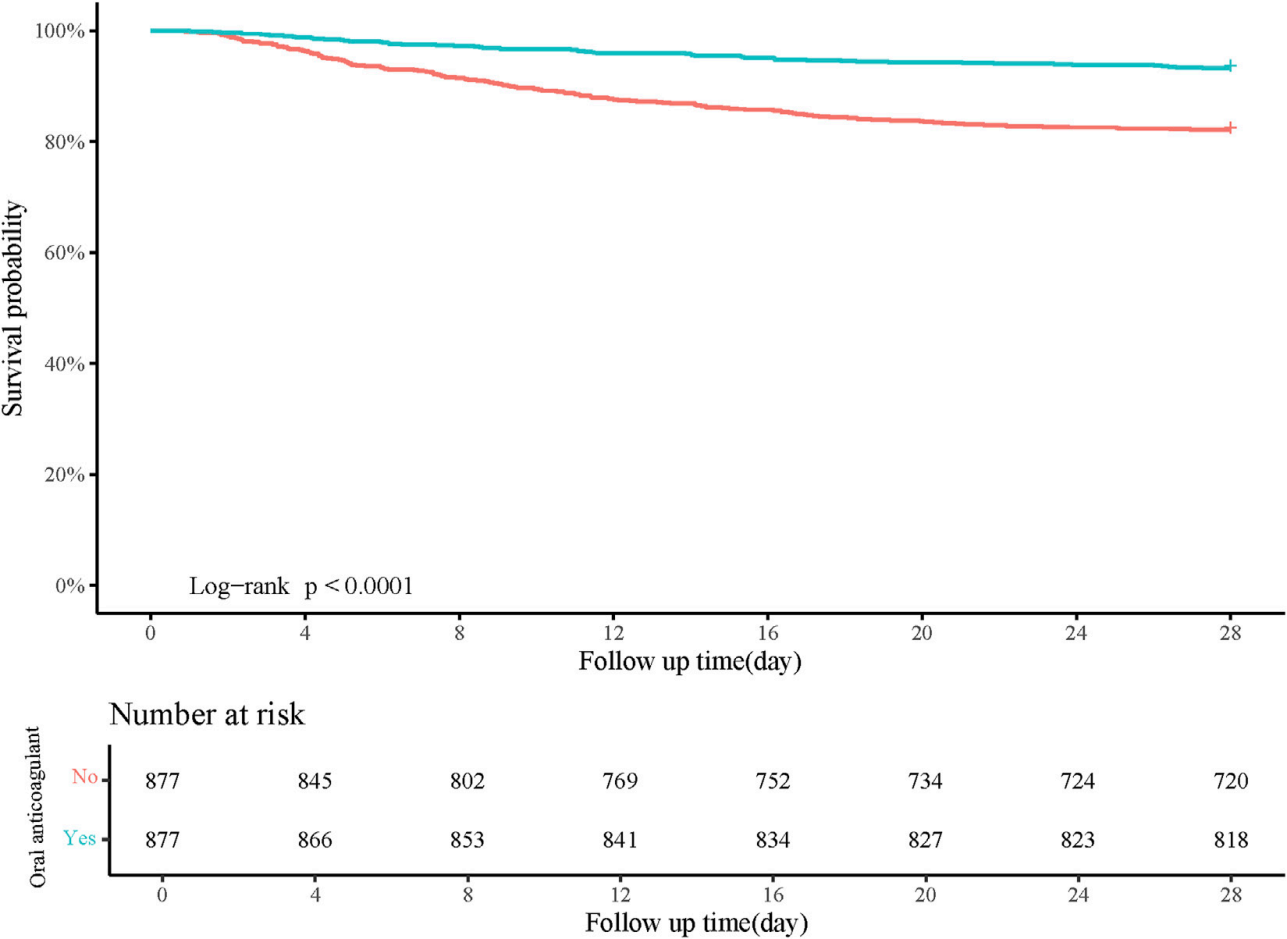
Kaplan-Meier curve of 28-day survival probability of patients receiving oral anticoagulant or not.

**FIGURE 4:**
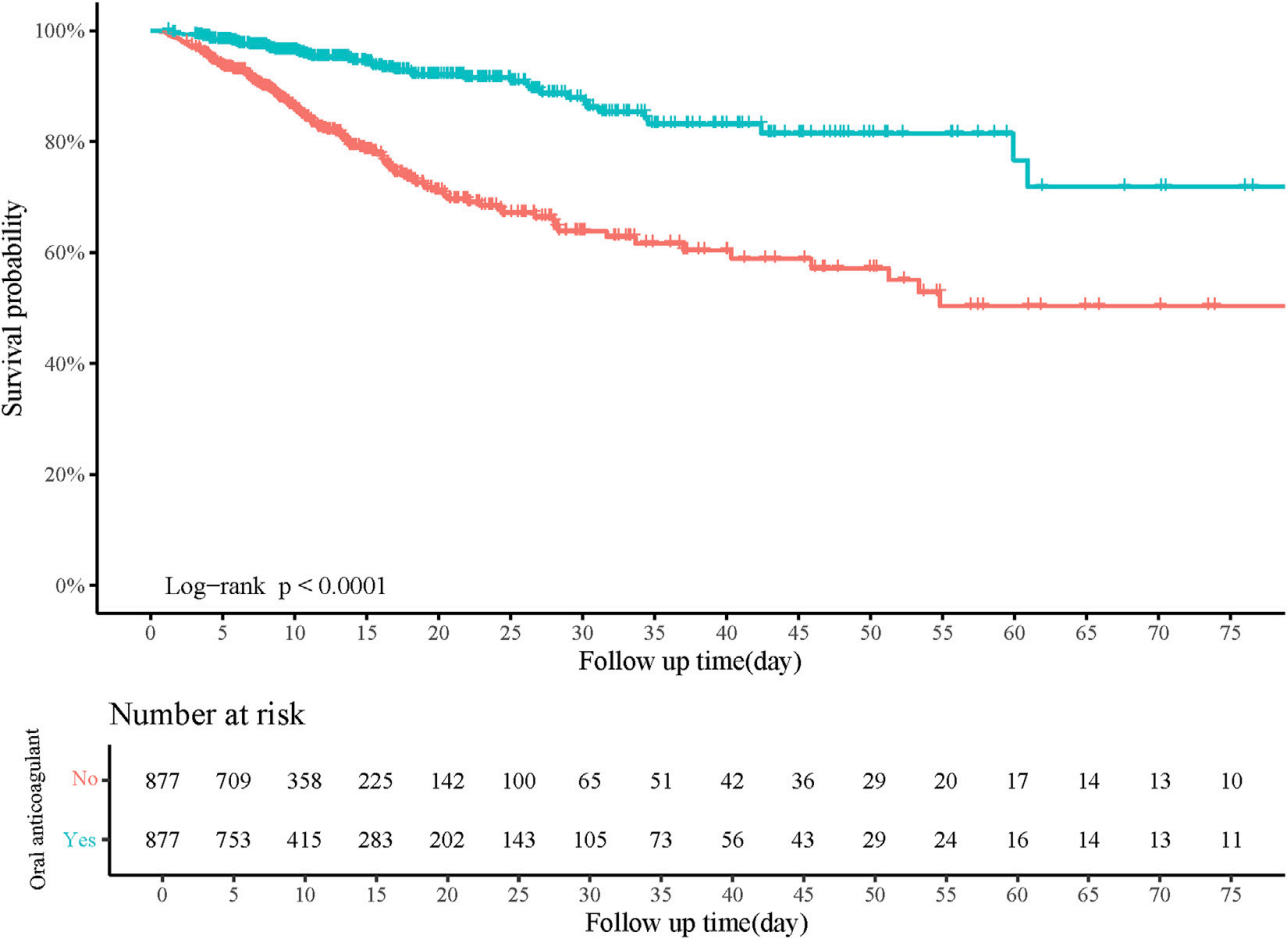
Kaplan-Meier curve of in-hospital survival probability of patients receiving oral anticoagulant or not.

Age, gender, pneumonia, AKI, heart rate, respiratory rate, SOFA, CCI, WBC, RDW, creatinine, BUN, anion gap, PTT, and RRT were confounding factors associated with in-hospital mortality (Table 2). The risk of in-hospital mortality might be decreased in patients who had oral anticoagulant in comparison with those did not have (HR = 0.29, 95%CI: 0.22–0.40). In the adjusted model, the association of oral anticoagulant use with reduced risk of in-hospital mortality was significant (HR = 0.27, 95%CI: 0.20–0.37) (Table 3). The 28-day survival probability of patients receiving oral anticoagulant was higher than those without oral anticoagulant use (Figure 3). ARDS patients had oral anticoagulant showed higher in-hospital survival probability than patients who did not use oral anticoagulant (Figure 4).

**TABLE 3:** Association between oral anticoagulant use with the risk of 28-day and in-hospital mortality (after PSM).

Outcomes	Variable	Model 1		Model 2	
		HR (95%CI)	*P*	HR (95%CI)	*P*
28-day mortality	Oral anticoagulant				
	No	Ref		Ref	
	Yes	0.35 (0.26–0.47)	<0.001	0.32 (0.24–0.44)	<0.001
In-hospital mortality	Oral anticoagulant				
	No	Ref		Ref	
	Yes	0.29 (0.22–0.40)	<0.001	0.27 (0.20–0.37)	<0.001

PSM: propensity score matching, Ref: reference, HR: hazard ratio, CI: confidence interval.

Model 1: not adjusted.

Model 2: 28-day mortality adjusted for gender, Ethnicity, ARDS, severity, cerebral infarction, pneumonia, sepsis; AKI, heart rate, respiratory rate, SOFA, CCI, WBC, creatinine; BUN, glucose, anion gap, SpO_2_, PTT, mechanical ventilation duration, antibiotics, and RRT.

In-hospital mortality adjusted for age, gender, pneumonia, AKI, heart rate, respiratory rate, SOFA, CCI, WBC, RDW, creatinine; BUN, anion gap; PTT, and RRT.

### Subgroup analysis on the association between oral anticoagulant use with the risk of 28-day and in-hospital mortality

The risk of 28-day mortality was identified to be decreased in both the ARDS mild (HR = 0.31, 95%CI: 0.16–0.58) or moderate/severe (HR = 0.33, 95%CI: 0.23–0.47) patients who received oral anticoagulant. In both the ARDS mild group (HR = 0.34, 95%CI: 0.18–0.65) and the moderate/severe group (HR = 0.25, 95%CI: 0.18–0.36), the risk of in-hospital mortality was reduced in patients with oral anticoagulant use. In ARDS patients complicated with sepsis or not, the risk of 28-day mortality and in-hospital mortality was decreased. Also, regardless ARDS patients complicated with atrial fibrillation or not, the risk of 28-day mortality and in-hospital mortality was decreased in those with oral anticoagulant use (Table 4).

**TABLE 4:** Subgroup analysis of the association between oral anticoagulant use with the risk of 28-day and in-hospital mortality (after PSM).

Outcomes	Variable	ARDS (mild) (n = 619)		ARDS (moderate/severe) (n = 1,135)	
		HR (95%CI)	*P*	HR (95%CI)	*P*
28-day mortality	Oral anticoagulant				
	No	Ref		Ref	
	Yes	0.31 (0.16–0.58)	<0.001	0.33 (0.23–0.47)	<0.001
In-hospital mortality	Oral anticoagulant				
	No	Ref		Ref	
	Yes	0.34 (0.18–0.65)	0.001	0.25 (0.18–0.36)	<0.001

Outcomes	Variable	Sepsis (no) (n = 944)		Sepsis (yes) (n = 810)	
		HR (95%CI)	*P*	HR (95%CI)	*P*
28-day mortality	Oral anticoagulant				
	No	Ref		Ref	
	Yes	0.44 (0.28–0.68)	<0.001	0.21 (0.13–0.34)	<0.001
In-hospital mortality	Oral anticoagulant				
	No	Ref		Ref	
	Yes	0.34 (0.22–0.54)	<0.001	0.17 (0.11–0.28)	<0.001

Outcomes	Variable	Atrial fibrillation (no) (n = 677)		Atrial fibrillation (yes) (n = 1,077)	
		HR (95%CI)	*P*	HR (95%CI)	*P*
28-day mortality	Oral anticoagulant				
	No	Ref		Ref	
	Yes	0.27 (0.15–0.46)	<0.001	0.34 (0.23–0.50)	<0.001
In-hospital mortality	Oral anticoagulant				
	No	Ref		Ref	
	Yes	0.28 (0.17–0.48)	<0.001	0.25 (0.17–0.37)	<0.001

PSM: propensity score matching, Ref: reference, HR: hazard ratio, CI: confidence interval.

Model 1: not adjusted.

Model 2: if not stratified.

28-day mortality adjusted for gender, Ethnicity, ARDS, severity, cerebral infarction, pneumonia, sepsis; AKI, heart rate, respiratory rate, SOFA, CCI, WBC, creatinine; BUN, glucose, anion gap, SpO_2_, PTT, mechanical ventilation duration, antibiotics, and RRT.

In-hospital mortality adjusted for age, gender, pneumonia, AKI, heart rate, respiratory rate, SOFA, CCI, WBC, RDW, creatinine; BUN, anion gap; PTT, and RRT.

### Comparisons of the effect of warfarin with novel oral anticoagulants on the risk of 28-day and in-hospital mortality

In total, 777 patients receiving warfarin, and 116 patients receiving novel oral anticoagulants. The percentage of participants receiving mechanical ventilation duration ≥48 h was lower in warfarin group relative to novel oral anticoagulants group (62.93% vs. 78.45%). The percentage of patients receiving antibiotics was higher in warfarin group than novel oral anticoagulants group (91.63% vs. 68.97%). Detailed information on the characteristics of patients receiving warfarin and novel oral anticoagulants was presented in Supplementary Table S3.

According to the data in Supplementary Table S4, age, weight, ARDS severity, cerebral infarction, pneumonia, heart rate, respiratory rate, CCI, RDW, creatinine, BUN, anion gap, SpO_2_, PaCO_2_, and RRT were covariates related to 28-day mortality. Age, gender, weight, pneumonia, sepsis, heart rate, respiratory rate, CCI, RDW, BUN, and anion gap were covariates related to in-hospital mortality. In the crude model, Warfarin might be related to decreased risk of 28-day mortality (HR = 0.35, 95%CI: 0.20–0.61) and in-hospital mortality (HR = 0.53, 95%CI: 0.30–0.95). After adjusting for the respective confounding factors, the associations of Warfarin with decreased risk of 28-day and in-hospital mortality were not significant (*P* > 0.05) (Table 5).

**TABLE 5:** Comparisons of the effect of warfarin with novel oral anticoagulants on the risk of 28-day and in-hospital mortality.

Outcomes	Variables	Model 1		Model 2	
		HR (95%CI)	*P*	HR (95%CI)	*P*
28-day mortality	Oral anticoagulant				
	New	Ref		Ref	
	Warfarin	0.35 (0.20–0.61)	<0.001	0.58 (0.30–1.12)	0.105
In-hospital mortality	Oral anticoagulant				
	New	Ref		Ref	
	Warfarin	0.53 (0.30–0.95)	0.033	0.69 (0.36–1.35)	0.282

PSM: propensity score matching, Ref: reference, HR: hazard ratio, CI: confidence interval.

Model 1: not adjusted.

Model 2: if not stratified.

28-day mortality adjusted for age, weight, ARDS, severity, cerebral infarction, pneumonia, heart rate, respiratory rate, CCI, RDW, creatinine; BUN, anion gap, SpO_2_, PaCO_2_, and RRT.

In-hospital mortality adjusted for age, gender, weight, pneumonia, sepsis, heart rate, respiratory rate, CCI, RDW, BUN, and anion gap.

## Discussion

The present study evaluated the association between oral anticoagulant use and 28-day and in-hospital mortality in patients with ARDS. We also compared the effects of warfarin and novel oral anticoagulants on 28-day and in-hospital mortality in patients with ARDS. The results indicated that oral anticoagulant use was related to reduced 28-day and in-hospital mortality risk in patients with ARDS. No significant difference on the 28-day and in-hospital mortality risk was observed between patients receiving warfarin and novel oral anticoagulants. The findings might provide a reference for making future medication strategies to improve the prognosis of ARDS patients.

Previously, increasing evidence has demonstrated the effect of anticoagulation administration on several lung-related diseases. Tang et al. revealed that anticoagulation therapy mainly with low molecular weight heparin was correlated with a reduced risk of mortality in patients with severe forms of coronavirus disease 2019 (COVID-19) (Tang et al., 2020). In hospitalized COVID-19 patients in the absence of contraindications, pharmacological antithrombotic prophylaxis was recommended (Thachil et al., 2020; American Society of Hematology, 2025). Nebulised unfractionated heparin was found to have strong scientific and biological rationale of its therapeutic potential, for patients with COVID‐19 requiring invasive mechanical ventilation (van Haren et al., 2022). Also, in patients with or at risk of ARDS, nebulised heparin was well tolerated and exploratory outcomes suggest less progression of lung injury and earlier return home (Dixon et al., 2021). The lungs of ARDS patients are characterized by inflammation and elevated levels of procoagulant factors, the absence of hydrostatic pulmonary edema, and disruption of the alveolar-capillary barrier, leading to increased permeability of proteins (Ware and Matthay, 2000; Gonzales et al., 2015). This leads to the activation of pulmonary macrophages toward a proinflammatory phenotype, an increase in both intravascular and extravascular neutrophils, platelets, and fibrin, as well as endothelial and epithelial damage (Camprubí-Rimblas et al., 2018). The severe inflammatory response and disseminated intravascular coagulation, in conjunction with virus-induced local inflammatory reactions, impairing endothelial cell function, results in vessel wall damage and subsequent microvascular thrombosis (Scudiero et al., 2021). Functional implications encompass a progressively deteriorating ventilation/perfusion mismatch and a diminished hypoxic pulmonary vasoconstriction reflex, accompanied by a significant component of microvascular pulmonary thrombosis (Ciceri et al., 2020). This mechanism, primarily characterized by endothelial injury and microvascular thrombosis, indicates that microvascular obstructive thrombo-inflammatory syndrome in the lungs may represent an atypical form of ARDS in patients with COVID-19 (Ciceri et al., 2020). Pulmonary coagulopathy in the pathophysiology of ARDS is characterized by an activation of the coagulation system and a concurrent reduction in fibrinolytic activity (Schultz et al., 2004; Tuinman et al., 2012). Various pathways of the coagulation cascade, such as the tissue factor (TF) pathway, the protein C pathway, and the regulation of fibrinolysis through the plasminogen activator (PA) and inhibitor pathways, play crucial roles in the pathophysiology of ARDS (Camprubí-Rimblas et al., 2018). The pathophysiological roles of the coagulation and fibrinolysis systems in ARDS have been extensively investigated through both experimental and clinical studies (Sonobe et al., 2023). Patients with coagulopathy exhibited adverse outcomes and persistent hypoxemia, indicating a significant association between coagulopathy and the development of ARDS. Given the established association between coagulopathy and inflammation as reported in prior studies, ARDS accompanied by coagulopathy may manifest as a hyperinflammatory phenotype. This condition is linked to multiple organ dysfunction syndrome and adverse outcomes (Shankar-Hari et al., 2019; Gando et al., 2019). Pulmonary and extrapulmonary microvascular thrombosis may significantly contribute to the development of acute lung injury and multiple organ dysfunction (Russo et al., 2020). In the present study, oral anticoagulant was identified to be related to more favorable short-term prognosis.

In addition, we found that novel oral anticoagulants had similar effect with warfarin on the risk of 28-day and in-hospital mortality. Previous evidence has suggested that the incidence of major and minor bleeding complications among patients in the emergency department who are on novel oral anticoagulants is comparable to that of patients receiving warfarin therapy as no significant differences between the warfarin and novel oral anticoagulants groups in the frequency of minor bleeding complications, major bleeding complications, and intracranial bleeding were identified (Dogan et al., 2022). Anderson et al. demonstrated that both novel oral anticoagulants and warfarin are safe and effective options for anticoagulation in patients experiencing postoperative atrial fibrillation (POAF) following coronary artery bypass grafting (CABG), and neither group had a major bleeding event during the initial hospitalization (Anderson et al., 2015).

This study explored the relationship between the use of oral anticoagulants and the risk of short-term mortality in patients with ARDS, which provided a reference for the formulation of medication strategies to improve the prognosis of ARDS patients. The study population was based on a large sample of critically ill patients with MIMICIV, and a variety of possible confounding factors including comorbidities, laboratory indicators, treatment and so on were controlled by PSM. There were several limitations. Firstly, this was a retrospective cohort study, the potential recall bias cannot be avoided. Secondly, the study population was from a single center in the United States, and the generalization of the results to other population should be conducted with caution. Thirdly, the total dose of patients receiving oral anticoagulant could not be calculated based on the data from the MIMIC-IV database, and the results from our study still requires verification in more studies. Fourthly, the number of patients with COVID ARDS was limited (n = 2), and whether COVID-9 caused ARDS could not be future analyzed. Fifthly, the cause of ARDS was not specific, which might affect the results. The association between oral anticoagulants and short-term prognosis in patients with ARDS still needs further validation in randomized controlled trials.

## Conclusions

The association between oral anticoagulant use and 28-day/in-hospital mortality in patients with ARDS was explored in the current study. The results delineated that oral anticoagulant was related to decreased risk of 28-day/in-hospital mortality in patients with ARDS. Warfarin and novel oral anticoagulants showed no significant difference on 28-day/in-hospital mortality in patients with ARDS. The findings might provide a reference for making future medical strategies for ARDS to improve the prognosis of these patients.

## Data Availability

Publicly available datasets were analyzed in this study. This data can be found here: MIMIC-IV database, https://mimic.physionet.org/iv/.
