# Supplementary material for: Roles of oral anticoagulant use on the risk of 28-day mortality and in-hospital mortality in patients with acute respiratory distress syndrome
Source: Front Pharmacol. 2025 May 14;16:1565312. doi: 10.3389/fphar.2025.1565312 (PMC12117219; doi:10.3389/fphar.2025.1565312)
Supplement: Supplementary file 1 [file Supplementaryfile1.docx]

Supplementary Table 1 The missing values of variables analyzed

| Variables | n | % |
| --- | --- | --- |
| Weight (kg) | 1060 | 12.24 |
| Heart rate (bpm) | 9 | 0.10 |
| Respiratory rate (insp/min) | 818 | 9.44 |
| Temperature (Deg.C) | 521 | 6.01 |
| SpO_2_ (%) | 10 | 0.12 |
| PaCO_2_ (mmHg) | 57 | 0.66 |
| White blood cell count (K/uL) | 79 | 0.91 |
| Platelet count (K/uL) | 77 | 0.89 |
| RDW (%) | 82 | 0.95 |
| Hematocrit (%) | 72 | 0.83 |
| Creatinine (mg/dL) | 66 | 0.76 |
| BUN (mg/dL) | 67 | 0.77 |
| Glucose (mg/dL) | 62 | 0.72 |
| Anion gap (mEq/L) | 87 | 1.00 |
| Calcium (mg/dL) | 1450 | 16.74 |
| PTT (sec) | 698 | 8.06 |
| Mean arterial pressure (mmHg) | 13 | 0.15 |
| Mechanical ventilation duration | 116 | 1.34 |

SpO_2_: oxygen saturation, PaCO_2_: partial pressure of carbon dioxide in arterial blood, RDW: red blood cell volume distribution width, BUN: blood urea nitrogen, PTT: partial thromboplastin time

Supplementary Table Sensitivity analysis of data before and after missing value imputation

| Variables | After imputation | Before imputation | Statistics | *P* |
| --- | --- | --- | --- | --- |
| Weight (kg), Mean ± SD | 87.79 ± 25.31 | 87.67 ± 25.39 | t=0.31 | 0.755 |
| Heart rate (bpm), Mean ± SD | 88.78 ± 19.79 | 88.76 ± 19.78 | t=0.05 | 0.961 |
| Respiratory rate (insp/min), M (Q_1_, Q_3_) | 17.00 (15.00, 22.00) | 18.00 (15.00, 22.00) | Z=1.302 | 0.193 |
| Temperature (Deg.C), Mean ± SD | 36.57 ± 1.04 | 36.58 ± 1.03 | t=-0.90 | 0.366 |
| SpO_2_ (%), Mean ± SD | 97.49 ± 4.38 | 97.49 ± 4.38 | t=0.02 | 0.983 |
| PaCO_2_ (mmHg), Mean ± SD | 43.43 ± 11.40 | 43.42 ± 11.39 | t=0.04 | 0.968 |
| White blood cell count (K/uL), M (Q_1_, Q_3_) | 12.50 (8.90, 16.80) | 12.50 (8.90, 16.80) | Z=0.074 | 0.941 |
| Platelet count (K/uL), M (Q_1_, Q_3_) | 169.00 (124.00, 233.00) | 169.00 (124.00, 233.00) | Z=-0.060 | 0.952 |
| RDW (%), Mean ± SD | 14.51 ± 2.04 | 14.51 ± 2.04 | t=0.08 | 0.934 |
| Hematocrit (%), Mean ± SD | 32.82 ± 6.91 | 32.81 ± 6.90 | t=0.08 | 0.940 |
| Creatinine (mg/dL), M (Q_1_, Q_3_) | 0.90 (0.70, 1.30) | 0.90 (0.70, 1.30) | Z=-0.251 | 0.802 |
| BUN (mg/dL), M (Q_1_, Q_3_) | 17.00 (13.00, 26.00) | 17.00 (13.00, 25.00) | Z=-0.142 | 0.887 |
| Glucose (mg/dL), M (Q_1_, Q_3_) | 140.00 (115.00, 176.00) | 140.00 (115.00, 176.00) | Z=-0.065 | 0.949 |
| Anion gap (mEq/L), M (Q_1_, Q_3_) | 13.00 (11.00, 16.00) | 13.00 (11.00, 16.00) | Z=0.102 | 0.919 |
| Calcium (mg/dL), Mean ± SD | 8.11 ± 0.94 | 8.11 ± 0.94 | t=-0.43 | 0.667 |
| PTT (sec), M (Q_1_, Q_3_) | 30.40 (27.00, 36.40) | 30.40 (27.00, 36.40) | Z=0.163 | 0.871 |
| Mean arterial pressure (mmHg), Mean ± SD | 83.90 ± 17.43 | 83.91 ± 17.44 | t=-0.03 | 0.979 |
| Mechanical ventilation duration (hours), n (%) |  |  | χ^2^=0.000 | 0.993 |
| <48 | 4222 (48.74) | 4166 (48.74) |  |  |
| ≥48 | 4441 (51.26) | 4381 (51.26) |  |  |

t: t-test, Z: Mann-Whitney U test, SD: standard deviation, M: Median, Q_1_:1st Quartile, Q_3_:3st Quartile, SpO_2_: oxygen saturation, PaCO_2_: partial pressure of carbon dioxide in arterial blood, RDW: red blood cell volume distribution width, BUN: blood urea nitrogen, PTT: partial thromboplastin time

Supplementary Table 3 The characteristics of participants receiving Warfarin or novel oral anticoagulants

| Variables | Total (n=893) | Novel oral anticoagulants (n=116) | Warfarin (n=777) | Statistics | *P* |
| --- | --- | --- | --- | --- | --- |
| Age (years), Mean ± SD | 64.57 ± 14.01 | 64.60 ± 12.66 | 64.56 ± 14.21 | t=0.03 | 0.977 |
| Gender, n (%) |  |  |  | χ^2^=0.359 | 0.549 |
| Female | 299 (33.48) | 36 (31.03) | 263 (33.85) |  |  |
| Male | 594 (66.52) | 80 (68.97) | 514 (66.15) |  |  |
| Ethnicity, n (%) |  |  |  | χ^2^=8.769 | 0.012 |
| White | 633 (70.88) | 69 (59.48) | 564 (72.59) |  |  |
| Black | 43 (4.82) | 9 (7.76) | 34 (4.38) |  |  |
| Other | 217 (24.30) | 38 (32.76) | 179 (23.04) |  |  |
| Weight (kg), Mean ± SD | 92.02 ± 26.41 | 90.44 ± 28.52 | 92.25 ± 26.09 | t=-0.69 | 0.491 |
| ARDS severity, n (%) |  |  |  | χ^2^=20.047 | <0.001 |
| Mild | 328 (36.73) | 24 (20.69) | 304 (39.12) |  |  |
| Moderate | 251 (28.11) | 50 (43.10) | 201 (25.87) |  |  |
| Severe | 314 (35.16) | 42 (36.21) | 272 (35.01) |  |  |
| Atrial fibrillation, n (%) |  |  |  | χ^2^=0.216 | 0.642 |
| No | 333 (37.29) | 41 (35.34) | 292 (37.58) |  |  |
| Yes | 560 (62.71) | 75 (64.66) | 485 (62.42) |  |  |
| Diabetes, n (%) |  |  |  | χ^2^=0.729 | 0.393 |
| No | 638 (71.44) | 79 (68.10) | 559 (71.94) |  |  |
| Yes | 255 (28.56) | 37 (31.90) | 218 (28.06) |  |  |
| Cerebral infarction, n (%) |  |  |  | χ^2^=1.205 | 0.272 |
| No | 885 (99.10) | 116 (100.00) | 769 (98.97) |  |  |
| Yes | 8 (0.90) | 0 (0.00) | 8 (1.03) |  |  |
| Pneumonia, n (%) |  |  |  | χ^2^=59.880 | <0.001 |
| No | 721 (80.74) | 63 (54.31) | 658 (84.68) |  |  |
| Yes | 172 (19.26) | 53 (45.69) | 119 (15.32) |  |  |
| Sepsis, n (%) |  |  |  | χ^2^=0.009 | 0.923 |
| No | 481 (53.86) | 62 (53.45) | 419 (53.93) |  |  |
| Yes | 412 (46.14) | 54 (46.55) | 358 (46.07) |  |  |
| AKI, n (%) |  |  |  | χ^2^=0.391 | 0.532 |
| No | 75 (8.40) | 8 (6.90) | 67 (8.62) |  |  |
| Yes | 818 (91.60) | 108 (93.10) | 710 (91.38) |  |  |
| Heart rate (bpm), Mean ± SD | 85.85 ± 17.53 | 91.59 ± 17.87 | 85.00 ± 17.32 | t=3.81 | <0.001 |
| Mean arterial pressure (mmHg), Mean ± SD | 81.99 ± 16.37 | 86.89 ± 18.23 | 81.26 ± 15.96 | t=3.15 | 0.002 |
| Respiratory rate (insp/min), M (Q_1_, Q_3_) | 16.00 (14.00, 20.00) | 20.00 (16.00, 25.00) | 16.00 (14.00, 19.00) | Z=7.926 | <0.001 |
| Temperature (Deg.C), Mean ± SD | 36.47 ± 0.93 | 36.79 ± 0.93 | 36.43 ± 0.92 | t=3.92 | <0.001 |
| SOFA, M (Q_1_, Q_3_) | 3.00 (1.00, 5.00) | 3.00 (0.50, 5.00) | 3.00 (1.00, 5.00) | Z=-0.452 | 0.652 |
| CCI, M (Q_1_, Q_3_) | 2.00 (1.00, 3.00) | 2.00 (1.00, 3.00) | 2.00 (1.00, 3.00) | Z=1.678 | 0.093 |
| White blood cell count (K/uL), M (Q_1_, Q_3_) | 13.00 (9.70, 17.00) | 12.00 (9.65, 15.55) | 13.10 (9.70, 17.00) | Z=-1.484 | 0.138 |
| Platelet count (K/uL), M (Q_1_, Q_3_) | 159.00 (123.00, 221.00) | 196.50 (134.50, 264.50) | 157.00 (122.00, 210.00) | Z=3.269 | 0.001 |
| RDW (%), Mean ± SD | 14.44 ± 1.75 | 14.85 ± 2.21 | 14.38 ± 1.66 | t=2.20 | 0.030 |
| Hematocrit (%), Mean ± SD | 32.09 ± 6.75 | 33.98 ± 7.18 | 31.81 ± 6.64 | t=3.25 | 0.001 |
| Creatinine (mg/dL), M (Q_1_, Q_3_) | 0.90 (0.70, 1.30) | 1.00 (0.80, 1.50) | 0.90 (0.70, 1.20) | Z=1.825 | 0.068 |
| BUN (mg/dL), M (Q_1_, Q_3_) | 17.00 (12.00, 25.00) | 20.00 (13.00, 31.50) | 17.00 (12.00, 23.00) | Z=2.018 | 0.044 |
| Glucose (mg/dL), M (Q_1_, Q_3_) | 142.00 (116.00, 171.00) | 146.00 (114.50, 173.00) | 141.00 (116.00, 171.00) | Z=0.694 | 0.488 |
| Anion gap (mEq/L), M (Q_1_, Q_3_) | 12.87 ± 4.13 | 12.97 ± 4.19 | 12.86 ± 4.12 | t=0.28 | 0.781 |
| Calcium (mg/dL), Mean ± SD | 8.16 ± 0.84 | 8.16 ± 0.81 | 8.16 ± 0.84 | t=0.02 | 0.988 |
| SPO2 (%), Mean ± SD | 97.65 ± 4.07 | 95.84 ± 4.70 | 97.92 ± 3.90 | t=-4.55 | <0.001 |
| PaCO2 (mmHg), Mean ± SD | 44.08 ± 11.85 | 45.84 ± 13.68 | 43.82 ± 11.54 | t=1.52 | 0.132 |
| PTT (sec), M (Q_1_, Q_3_) | 31.50 (27.70, 38.50) | 29.70 (27.20, 38.05) | 31.70 (27.90, 38.50) | Z=-1.341 | 0.180 |
| Mechanical ventilation duration (hours), n (%) |  |  |  | χ^2^=10.671 | 0.001 |
| <48 | 313 (35.05) | 25 (21.55) | 288 (37.07) |  |  |
| ≥48 | 580 (64.95) | 91 (78.45) | 489 (62.93) |  |  |
| Vasopressors, n (%) |  |  |  | χ^2^=8.103 | 0.004 |
| No | 241 (26.99) | 44 (37.93) | 197 (25.35) |  |  |
| Yes | 652 (73.01) | 72 (62.07) | 580 (74.65) |  |  |
| Antibiotics, n (%) |  |  |  | χ^2^=51.707 | <0.001 |
| No | 101 (11.31) | 36 (31.03) | 65 (8.37) |  |  |
| Yes | 792 (88.69) | 80 (68.97) | 712 (91.63) |  |  |
| Heparin, n (%) |  |  |  | χ^2^=8.215 | 0.004 |
| No | 632 (70.77) | 69 (59.48) | 563 (72.46) |  |  |
| Yes | 261 (29.23) | 47 (40.52) | 214 (27.54) |  |  |
| RRT, n (%) |  |  |  | χ^2^=1.511 | 0.219 |
| No | 799 (89.47) | 100 (86.21) | 699 (89.96) |  |  |
| Yes | 94 (10.53) | 16 (13.79) | 78 (10.04) |  |  |
| In-hospital mortality, n (%) |  |  |  | χ^2^=19.391 | <0.001 |
| Alive | 837 (93.73) | 98 (84.48) | 739 (95.11) |  |  |
| Dead | 56 (6.27) | 18 (15.52) | 38 (4.89) |  |  |
| Follow-up of in-hospital mortality, M (Q_1_, Q_3_) | 9.21 (5.96, 18.16) | 16.75 (8.29, 30.35) | 8.98 (5.64, 16.15) | Z=5.121 | <0.001 |
| 28-day mortality, n (%) |  |  |  | χ^2^=13.398 | <0.001 |
| Alive | 833 (93.28) | 99 (85.34) | 734 (94.47) |  |  |
| Dead | 60 (6.72) | 17 (14.66) | 43 (5.53) |  |  |
| Follow-up of 28-day mortality, M (Q_1_, Q_3_) | 28.00 (28.00, 28.00) | 28.00 (28.00, 28.00) | 28.00 (28.00, 28.00) | Z=-3.684 | <0.001 |

t: t-test, Z: Mann-Whitney U test, χ^2^: Chi-square test

SD: standard deviation, M: Median, Q_1_:1st Quartile, Q_3_:3st Quartile, ARDS: acute respiratory distress syndrome, AKI: acute kidney injury, SOFA: sepsis related organ failure assessment, CCI: Charlson comorbidity index, RDW: red blood cell volume distribution width, BUN: blood urea nitrogen, SpO_2_: oxygen saturation, PaCO_2_: partial pressure of carbon dioxide in arterial blood, PTT: partial thromboplastin time, RRT: renal replacement therapy

Supplementary Table Potential confounding factors associated with 28-day mortality and in-hospital mortality (n=893)

| Variables | 28-day mortality | | In-hospital mortality | |
| --- | --- | --- | --- | --- |
|  | HR (95%CI) | *P* | HR (95%CI) | *P* |
| Age (years) | 1.04 (1.02-1.06) | <0.001 | 1.05 (1.02-1.07) | <0.001 |
| Gender |  |  |  |  |
| Female | Ref |  | Ref |  |
| Male | 0.63 (0.37-1.04) | 0.073 | 0.51 (0.30-0.87) | 0.014 |
| Ethnicity |  |  |  |  |
| White | Ref |  | Ref |  |
| Black | 1.77 (0.70-4.46) | 0.228 | 1.40 (0.55-3.59) | 0.479 |
| Other | 0.74 (0.38-1.43) | 0.369 | 0.76 (0.40-1.45) | 0.408 |
| Weight (kg) | 0.98 (0.97-0.99) | <0.001 | 0.97 (0.96-0.99) | <0.001 |
| ARDS severity |  |  |  |  |
| Mild | Ref |  | Ref |  |
| Moderate | 2.34 (1.24-4.41) | 0.009 | 1.41 (0.72-2.75) | 0.319 |
| Severe | 1.25 (0.63-2.48) | 0.521 | 0.85 (0.42-1.74) | 0.663 |
| Atrial fibrillation |  |  |  |  |
| No | Ref |  | Ref |  |
| Yes | 1.17 (0.68-2.00) | 0.577 | 1.06 (0.61-1.82) | 0.848 |
| Diabetes |  |  |  |  |
| No | Ref |  | Ref |  |
| Yes | 1.20 (0.69-2.06) | 0.521 | 0.91 (0.51-1.63) | 0.743 |
| Cerebral infarction |  |  |  |  |
| No | Ref |  | Ref |  |
| Yes | 6.88 (2.15-21.99) | 0.001 | 3.72 (0.90-15.40) | 0.070 |
| Pneumonia |  |  |  |  |
| No | Ref |  | Ref |  |
| Yes | 2.74 (1.62-4.63) | <0.001 | 1.79 (1.03-3.10) | 0.039 |
| Sepsis |  |  |  |  |
| No | Ref |  | Ref |  |
| Yes | 0.91 (0.54-1.52) | 0.721 | 0.49 (0.29-0.85) | 0.012 |
| AKI |  |  |  |  |
| No | Ref |  | Ref |  |
| Yes | - | 0.985 | - | 0.983 |
| Heart rate (bpm) | 1.02 (1.01-1.03) | 0.001 | 1.01 (1.01-1.02) | 0.014 |
| Mean arterial pressure (mmHg) | 1.01 (0.99-1.02) | 0.227 | 1.00 (0.99-1.02) | 0.808 |
| Respiratory rate (insp/min) | 1.07 (1.04-1.10) | <0.001 | 1.05 (1.02-1.09) | 0.002 |
| Temperature (Deg.C) | 1.29 (0.98-1.68) | 0.066 | 1.20 (0.92-1.56) | 0.172 |
| SOFA | 1.09 (1.00-1.20) | 0.053 | 1.05 (0.96-1.15) | 0.316 |
| CCI | 1.36 (1.23-1.51) | <0.001 | 1.21 (1.08-1.36) | 0.002 |
| White blood cell count (K/uL) | 0.97 (0.93-1.02) | 0.206 | 0.98 (0.94-1.02) | 0.342 |
| Platelet count (K/uL) | 1.00 (1.00-1.00) | 0.478 | 1.00 (1.00-1.00) | 0.782 |
| RDW (%) | 1.42 (1.30-1.55) | <0.001 | 1.36 (1.24-1.50) | <0.001 |
| Hematocrit (%) | 1.00 (0.97-1.04) | 0.896 | 0.99 (0.96-1.03) | 0.727 |
| Creatinine (mg/dL) | 1.20 (1.06-1.37) | 0.005 | 1.06 (0.90-1.24) | 0.493 |
| BUN (mg/dL) | 1.03 (1.02-1.04) | <0.001 | 1.02 (1.01-1.03) | <0.001 |
| Glucose (mg/dL) | 1.00 (1.00-1.01) | 0.070 | 1.00 (1.00-1.01) | 0.251 |
| Anion gap (mEq/L) | 1.09 (1.05-1.13) | <0.001 | 1.07 (1.02-1.12) | 0.003 |
| Calcium (mg/dL) | 0.92 (0.68-1.24) | 0.571 | 1.02 (0.76-1.35) | 0.916 |
| SPO2 (%) | 0.95 (0.91-0.99) | 0.024 | 0.96 (0.92-1.01) | 0.108 |
| PaCO2 (mmHg) | 0.97 (0.94-0.99) | 0.043 | 0.97 (0.95-1.00) | 0.058 |
| PTT (sec) | 1.00 (0.99-1.01) | 0.707 | 1.01 (1.00-1.02) | 0.072 |
| Mechanical ventilation duration (hours) |  |  |  |  |
| <48 | Ref |  | Ref |  |
| ≥48 | 1.22 (0.70-2.12) | 0.487 | 0.54 (0.28-1.07) | 0.079 |
| Vasopressors |  |  |  |  |
| No | Ref |  | Ref |  |
| Yes | 1.19 (0.65-2.17) | 0.572 | 1.07 (0.57-2.00) | 0.843 |
| Antibiotics |  |  |  |  |
| No | Ref |  | Ref |  |
| Yes | 0.69 (0.34-1.40) | 0.307 | 0.73 (0.35-1.50) | 0.390 |
| Heparin |  |  |  |  |
| No | Ref |  | Ref |  |
| Yes | 0.68 (0.36-1.25) | 0.211 | 0.58 (0.30-1.13) | 0.111 |
| RRT |  |  |  |  |
| No | Ref |  | Ref |  |
| Yes | 2.77 (1.52-5.05) | <0.001 | 1.28 (0.69-2.41) | 0.435 |

Ref: reference, HR: hazard ratio, CI: confidence interval, ARDS: acute respiratory distress syndrome, AKI: acute kidney injury, SOFA: sepsis related organ failure assessment, CCI: Charlson comorbidity index, RDW: red blood cell volume distribution width, BUN: blood urea nitrogen, SpO_2_: oxygen saturation, PaCO_2_: partial pressure of carbon dioxide in arterial blood, PTT: partial thromboplastin time, RRT: renal replacement therapy
